# A New Perspective for the Treatment of Alzheimer’s Disease: Exosome-like Liposomes to Deliver Natural Compounds and RNA Therapies

**DOI:** 10.3390/molecules28166015

**Published:** 2023-08-11

**Authors:** Joana Ribeiro, Ivo Lopes, Andreia Castro Gomes

**Affiliations:** 1 Centre of Molecular and Environmental Biology (CBMA)/Aquatic Research Network (ARNET) Associate Laboratory, Universidade do Minho, Campus de Gualtar, 4710-057 Braga, Portugal; pg41579@alunos.uminho.pt (J.R.); ivo_lopes_@hotmail.com (I.L.); 2 Institute of Science and Innovation for Sustainability (IB-S), Universidade do Minho, Campus de Gualtar, 4710-057 Braga, Portugal

**Keywords:** neurodegenerative diseases, Alzheimer’s disease, natural compounds, RNA therapy, blood-brain barrier, exosome-like liposomes

## Abstract

With the increment of the aging population in recent years, neurodegenerative diseases exert a major global disease burden, essentially as a result of the lack of treatments that stop the disease progression. Alzheimer’s Disease (AD) is an example of a neurodegenerative disease that affects millions of people globally, with no effective treatment. Natural compounds have emerged as a viable therapy to fill a huge gap in AD management, and in recent years, mostly fueled by the COVID-19 pandemic, RNA-based therapeutics have become a hot topic in the treatment of several diseases. Treatments of AD face significant limitations due to the complex and interconnected pathways that lead to their hallmarks and also due to the necessity to cross the blood–brain barrier. Nanotechnology has contributed to surpassing this bottleneck in the treatment of AD by promoting safe and enhanced drug delivery to the brain. In particular, exosome-like nanoparticles, a hybrid delivery system combining exosomes and liposomes’ advantageous features, are demonstrating great potential in the treatment of central nervous system diseases.

## 1. Introduction

Neurodegenerative diseases are a diversified group of conditions characterized by progressive degeneration of the function of the central nervous system (CNS) or peripheral nervous system, and the current therapeutic options do not provide a cure, only slowing down the disease progression. Together, neurodegenerative diseases exert a major burden in global healthcare systems, with dementia being a public health challenge in many developed countries, as aging is a strong risk factor [1].

Dementia is one of the highest global health crises of this century, with Alzheimer’s disease (AD) being the most common form of dementia. In the United States, an estimated 6.7 million individuals aged 65 and older are living with AD in 2023, and the number is expected to reach 88 million by 2050 [2]. The estimated 2023 cost of caring for those with this disease is $345 billion. Between 2000 and 2019, the number of deaths from AD increased by 145%, while deaths from the number-one cause of death—heart disease—decreased by 7.3% [2].

AD is a progressive, irreversible neurodegenerative disease that leads to memory impairment, impacts cognition, and can ultimately affects behavior, speech, visuospatial orientation, and the motor system. This disease is characterized by two major pathological hallmarks: Progressive accumulation of amyloid beta (Aβ) plaques and neurofibrillary tangles (NFTs). Aβ damages neurons by interfering with neuron communication at synapses and NFTs block the transport of essential molecules for the normal function of neurons. Consequently, these lead to other complications such as oxidative stress, inflammation, and brain atrophy due to cell loss [2,3].

Natural compounds or extracts are a viable therapy to fill the huge gap in the treatment of this disease since they can target several hallmarks. However, for improved efficacy of these compounds, they can be administered by delivery systems. Moreover, mostly instigated by the recent COVID-19 pandemic, RNA-based therapies have become a topic of great interest to researchers and pharmaceutical companies. RNA therapies promise to change the current conventional drugs that are not capable to target and treat all types of diseases, and several clinical studies are ongoing for a variety of RNA-based therapeutics against various incurable diseases. RNA therapy presents several advantages such as cost effectiveness, manufacturing simplicity, and the ability to target previously inaccessible pathways [4,5]. RNA therapies such as microRNAs (miRNAs), small interfering RNAs (siRNAs), and messenger RNAs (mRNAs) seem to be some of the most promising molecules for the treatment of AD [6]. For the effective delivery of RNA, this molecule has to overcome several obstacles. Its hydrophilic, negatively charged properties make it difficult for the RNA molecule to passively diffuse across the cell’s membrane, and so it has to undergo endocytosis and escape from the endosome to reach the cytoplasm. Furthermore, this molecule is highly susceptible to ribonucleases degradation and must have enhanced accumulation at targeted tissues [4].

In addition to the complex mechanisms that lead to AD, the blood–brain barrier (BBB) is known to be a particular reason for the lack of effective treatments for AD. The BBB is a physiological barrier constituted by blood vessels that vascularize the CNS and possess unique properties that allow precise control of the molecules allowed to enter the CNS [7].

Nanoparticles have been used to mitigate all of these hindrances in the delivery of RNA molecules into the brain. These drug delivery systems effectively protect RNA from degradation, enable the crossing of biological barriers, and allow a targeted accumulation and release [8]. In recent years, nanoparticles such as dendrimers, polymeric nanoparticles and gold nanoparticles, and carbon quantum dots have shown to be capable of crossing the BBB effectively [8]. Along with these nanoparticles, exosomes and liposomes are delivery systems with promising properties that allow them to cross the BBB. Exosomes are nano-sized extracellular vesicles (EVs) released into surrounding body fluids by their parental cells and carry cell-specific cargos of proteins, lipids, and genetic materials. These EVs can be selectively uptaken by neighboring or distant cells far from their release. On the other hand, liposomes are synthetic vesicles comprised of one or several concentric lipid bilayers surrounding an aqueous lumen that can be created from cholesterol and natural phospholipids or synthetic surfactants [9,10].

Exosome-like liposomes are a novel concept of nanoparticles that combine the advantages of both the exosomes and liposomes, creating a unique delivery system with several advantages such as the mimetic constituents of natural exosomes, high biocompatibility, small size, easy production, efficient transport and delivery of therapeutical compounds with low bioavailability (e.g., curcumin and RNA molecules), and the ability to load both hydrophilic and hydrophobic drugs [11].

## 2. Neurodegenerative Diseases

Neurodegenerative diseases are a heterogeneous group of neurological disorders characterized by cognitive, psychiatric, and motor deficits due to neuron loss [12]. In addition to these common features, there are also no current treatments to stop the advancement of the diseases. Some of the main reasons for the lack of effective treatment in neurodegenerative diseases are the limitations imposed by the BBB and the complex pathways that lead to the late diagnosis of the diseases [13].

**Figure 1:**
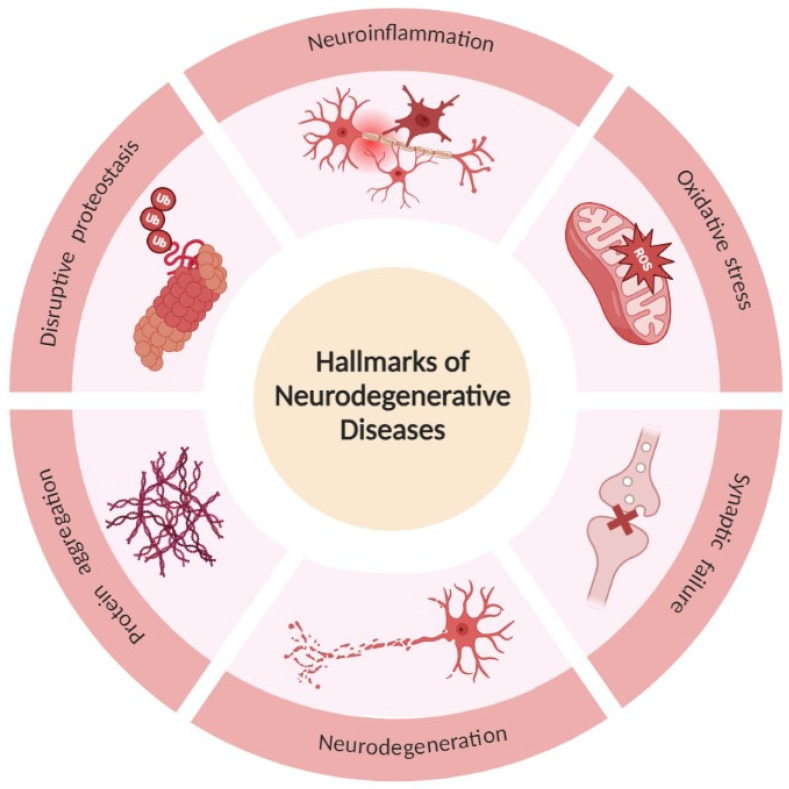
Hallmarks of neurodegenerative diseases. The scheme illustrates the six hallmarks of these disorders: Protein aggregation, disruptive proteostasis, neuroinflammation, oxidative stress, synaptic failure, and neuronal death. Created in BioRender.com (accessed on 25 May 2023).

Neurodegenerative diseases are characterized by (1) protein aggregation; (2) disruptive proteostasis; (3) neuroinflammation; (4) oxidative stress; (5) synaptic failure; and (6) neuronal death (Figure 1). The presence of protein aggregation is a key hallmark in a large variety of neurodegenerative disorders. These abnormally deposited proteins are found in brain regions that, when damaged, lead to physical vulnerability [1,14]. Several proteins are associated with neurodegenerative disorders:

- Tau protein—microtubule-associated protein—encoded by the microtubule-associated protein tau (MAPT) gene. Tau is substantially expressed in the cytoplasm of neurons and plays an important role primarily in the stabilization and assembly of axonal microtubules and also in a variety of physiological processes, which include axonal transport, signal transmission between neurons, neurogenesis, myelination, motor function, neuronal excitability, glucose metabolism, iron homeostasis, and DNA protection [15,16].
- Aβ—derives from the amyloid precursor protein (APP) and aggregates into amyloid plaques with Aβ polypeptides 40 and 42 amino acids long [17].
- Prion—another protein present in neurodegenerative diseases is the prion protein (PrP) encoded by the PRNP gene. In prion diseases, the prion protein misfolds, propagates, and aggregates rapidly, being responsible for spreading neurodegeneration between cells, and, consequently, brain regions [14].
- α-synuclein—this is a 140-amino-acid protein highly expressed in the brain, encoded by the α-synuclein (*SCNA*) gene [12].

Under healthy conditions, there are protein degradation systems to maintain protein homeostasis, with an important role in the clearance of toxic protein aggregates: The autophagy lysosomal pathway and the ubiquitin-proteosome system. However, these pathways lose activity in elderly individuals, contributing to the accumulation of toxic protein aggregates [14]. Glial cells (microglia and astrocyte) substitute peripheral immune cells’ function in the brain. Microglia play a crucial role in defense functions in the brain, and these cells are activated in signs of pathogens or injury. In neurotoxic conditions with several aggregated proteins, microglia activation is induced, interacting with astrocytes and leading to inflammation [14,18]. Neurons are particularly susceptible to oxidative stress due to the high polyunsaturated fatty acid content in the membranes, high oxygen consumption, and low antioxidant defenses in the brain, inducing increased oxidation of proteins, nucleic acids, and lipids [1]. Synaptic failure has been described in various neurodegenerative diseases. Synapses are the functional part of the connection between neurons and the key physiological function of neurons. However, when pathogenic factors affect synapses, it suppresses the brain from learning and leads to memory impairment. Currently, these synaptic changes are the targets of many pharmacological interventions [19]. All these factors result in neuronal cell death and, in some neurodegenerative disorders, can result in brain volume loss [14].

Neurodegenerative diseases include Parkinson’s disease (PD), amyotrophic lateral sclerosis (ALS), Huntington’s disease (HD), and AD, which will be the focus of this review [1].

The brain of PD patients is affected by the presence of intra-neuronal inclusion bodies—Lewy bodies—and the accumulation of the protein α-synuclein, which spreads from one brain region to another. PD is characterized by movement disorder, motor function impairment, and other nonmotor symptoms, such as gastrointestinal issues and sleep disturbances [1,20].

ALS is a devastating, progressive disease, and the cause of this condition is unknown. This disease is characterized by a deficit in motor neurons in the spinal cord and the motor cortex of the cerebrum. Patients witness progressive muscle weakness and atrophy and respiratory failure due to the weakening of respiratory muscles, with a life expectancy of 15.8 months post-diagnosis [21,22].

HD is an inherited neurodegenerative disorder caused by a mutation in the Huntingtin gene, an abnormal trinucleotide expansion, which is translated into a mutant protein. The protein leads to the disruption of cellular molecular processes, which can involve both loss- and gain-of-function mechanisms. A hallmark of this disease is the degeneration of the striatum (caudate nucleus and putamen), with specific loss of efferent medium spiny neurons and brain shrinkage. This disorder is characterized by movement disturbance, cognitive decline, coordination loss, depression, obsessive–compulsive disorder, and other psychiatric symptoms [23,24,25].

### Alzheimer’s Disease

**Figure 2:**
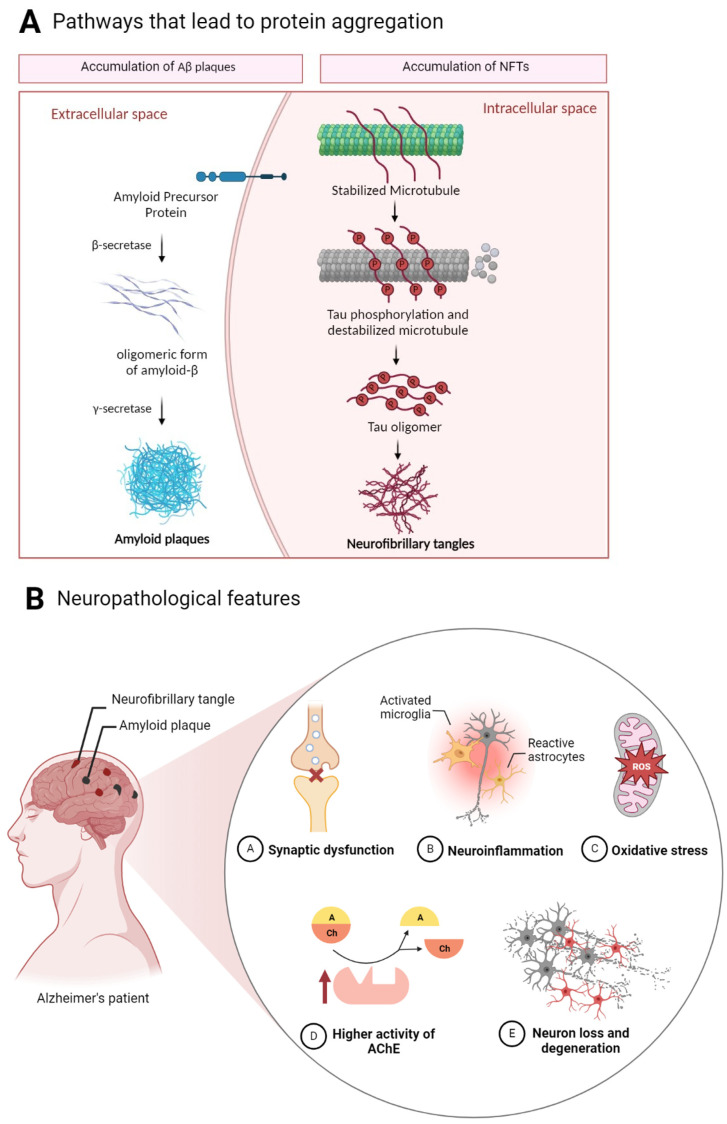
Alzheimer’s disease hallmarks. (**A**) Pathway that leads to the formation of amyloid plaques and the mechanism responsible for the formation of neurofibrillary tangles. (**B**) Accumulation of amyloid plaques and neurofibrillary tangles provoke disorders such as synaptic dysfunction, neuroinflammation, oxidative stress, and higher activity of acetylcholinesterase. These result in neurodegeneration. Created in BioRender.com (accessed on 20 April 2023).

The pathological development of AD is complex and not yet fully understood [1]. At the microscopic level, the progression of this disease is characterized by the accumulation of two proteins: Aβ and Tau proteins, which aggregate into Aβ plaques and NFTs, respectively (Figure 2A) [26]. 

The presence of amyloid plaques is a consequence of an abnormal accumulation and deposition of Aβ, a product of the amyloid precursor protein (APP) [27]. APP is a transmembrane protein expressed in numerous human tissues, including the CNS, and can be cleaved by different proteases in the non-amyloidogenic or amyloidogenic processing of APP [28,29]. If the APP is cleaved by α-secretase, in the non-amyloidogenic pathway, it prevents the formation of the Aβ and, instead, soluble APPα fragments are formed, which are described to be non-cytotoxic [26]. The amyloidogenic pathway includes the combined action of β- and γ- secretases, which generate Aβ peptides with different C-terminal residues (Aβ_40_ and Aβ_42_) [15,27]. The deposition and accumulation of these Aβ polypeptides lead to the formation of amyloid plaques in the brain, resulting in neuroinflammation and synaptic dysfunction [30,31,32]. NFTs are a consequence of the deposition of the abnormally phosphorylated Tau protein, leading to its aggregation [16]. The microtubule-binding protein Tau, a cytoskeletal protein produced by alternative splicing of the MAPT gene, has been identified as a key molecule in AD, but also in a series of neurodegenerative diseases referred to as tauopathies, in contrast to Aβ accumulation, which is a characteristic exclusive to AD [15,33,34]. Hyperphosphorylated Tau loses its functions in the synthesis and stabilization of microtubules. These proteins accumulate in neurites and neuronal cell bodies, where it develops into insoluble aggregates, NFTs. Tau can be secreted into the extracellular space either in its naked form or packaged in exosomes [35,36,37].

In addition the accumulation of these two proteins in the brain, there are other hallmarks in AD, such as synaptic impairment neuroinflammation, oxidative stress, and higher activity of acetylcholinesterase (AChE) (Figure 2B). Synaptic impairment is responsible for the reduction in presynaptic vesicle release and the decrease in glutamatergic receptors [33,38]. Additionally, microglia cells develop more susceptibility to stimulus and produce inflammatory cytokines and chemokines, leading to cytotoxic and pro-inflammatory events. Consequently, this contributes to the deterioration of the BBB and the inability to remove neurotoxic molecules such as Aβ plaques and NFTs from the CNS [39,40]. Damage to the mitochondrial structure, integrity, and biogenesis lead to excessive reactive oxygen species (ROS) production, causing damage to the cellular structure. The equilibrium of distinct neurotransmitter systems, such as acetylcholine (Ach), is crucial for healthy brain function [41,42]. In AD, alterations in the cholinergic system are often present, since there is a loss of cholinergic neurons that leads to an extensive decline of ACh, and consequently to a deficit of cholinergic transmission at the pre-synaptic level. This ACh decrease is due to the higher activity of AChE, the enzyme responsible for its degradation [43]. All of the mentioned microscopic hallmarks alter the brain at a macroscopic level, causing brain shrinkage with cortical thinning and atrophy, leading to decreased brain weight [3,27].

Currently, the therapeutical options for these patients are still very limited [44,45]. The main classes of drugs available to treat AD are acetylcholinesterase inhibitors, which include donepezil, rivastigmine, and galantamine. Although the equilibrium of different neurotransmitters, such as acetylcholine, plays a key role in normal brain function, the cholinergic system is not the only system affected by this pathology. Thus, cholinesterase inhibitors only treat symptoms and do not prevent disease progression. Treatments directed to other hallmarks, such as Aβ accumulation and Tau hyperphosphorylation, failed to provide effects [42,46]. Therefore, an innovative approach that can efficiently treat and control each hallmark of this multifactorial disease is required for effective AD treatment [47].

## 3. Natural Compounds for AD Treatment

Natural drugs are gaining increased interest from both scientific academia and pharmaceutical industries for the therapy of several diseases. In recent years, due to their multiple beneficial properties, more than 100 natural products have been proposed as a promising approach for AD therapy [48]. Many molecules, including lignans, flavonoids, tannins, polyphenols, triterpenes, sterols, and alkaloids, can act via different pathways since they have anti-inflammatory, anti-amyloidogenic, anticholinesterase, and anti-inflammatory properties and can reduce oxidative stress [49]. It is reasonable to speculate that the progression of AD could be slowed down or even prevented by natural products working on multiple pathological targets [50]. Table 1 presents an array of natural compounds or extracts that were researched for AD in the last fifteen years found on PubMed and Web of Science.

### Overcoming Limitations of Natural Compounds with Delivery Systems

**Figure 3:**
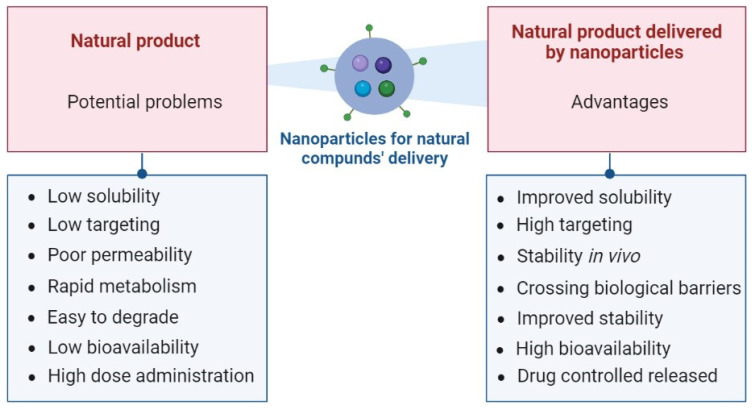
Potential problems of natural products in vivo and advantages of natural products delivered by nanoparticles. Created in BioRender.com (accessed on 5 May 2023).

In addition to the multiple beneficial properties of natural products, these display several limitations such as low hydrophilicity, rapid metabolism and degradation, low bioavailability, reduced targeting, susceptibility to physiological media, and poor permeability through lipid bilayers. Consequently, in vivo, natural drugs require a high-dose administration beyond a safe range, to result in an effective and safe bioavailability [48,79]. Nanotechnology represents a new method to overcome these challenges. Delivery systems for natural compounds lead to the enhancement of pharmacological activity by improving the stability of drugs in vivo, bioavailability, and controlled release, increasing the accumulation of active ingredients in target sites, promoting the solubility of insoluble drugs, and reducing the required doses to produce therapeutic effects [79]. Nanotechnology offers multiple advantages in the delivery of natural products, since by this method, these drugs can exert their therapeutic effect in the treatment of AD (Figure 3) [80].

## 4. RNAs as a Promising Tool in the Treatment of AD

The small-molecule- and protein-based therapies that interact with a particular biologic molecule to obtain a pharmacological response to control a disease have been successfully dealing with many diseases in past years [81]. However, this conventional pharmacotherapy has several limitations. Protein-based medications primarily target proteins to inhibit their activity, and only ~1.5% of the human genome encodes proteins. Consequently, the range of disease targets of this drug is limited and unable to meet the required demands [5,82]. Most protein-based drugs are too large to enter their target cells and therefore are only effective when their target molecule is extracellular, demonstrating difficult tissue penetration [82,83].

The study of RNA therapeutics started decades ago, leading to a long scientific journey. However, just recently, this field of research has developed dramatically as a result of the response to the COVID-19 pandemic, which revealed how RNA-based therapeutics could lead to a new era of different and accessible new technology in combatting a wide range of diseases [40,84]. In addition to the inherent instability of RNA, these molecules possess particular features and versatility, with multiple advantages over protein and DNA-based drugs. These characteristics include the inducement of protein coding, binding specificity to target molecules, and inhibition of protein translation. Additionally, RNA has the capacity to recognize a wide range of ligands, targeting almost any genetic component within the cell that is out of reach for the most established drug models [81,84].

RNA can be modified in the base, backbone, and sugar, increasing target affinity and preventing nuclease digestion [85]. These modifications make them completely different from cellular RNAs transcribed from the genome [81]. Thus, unlike DNA-based therapeutics, which must cross the cytoplasmic and nuclear membrane and can integrate the host genome and cause a mutation, RNA therapeutics have no risk of chromosomal integration, exhibiting a safer profile [5,86]. Another important advantage of RNA therapy is its long-lasting effects when using, for example, siRNAs as a drug, benefiting patients who cannot receive frequent treatments [87]. The fast production of RNA-based therapies is also a distinct advantage, considering the increased knowledge that will provide faster and easier design of RNA molecules when compared to the process to produce novel protein-based drugs, which takes years. The vaccine’s rapid production and successful reduction of the severity of the disease in infected people during the COVID-19 pandemic is evidence of how quickly this type of therapy can be developed and implemented [86].

A long scientific journey has led to prominent technological advances in the RNA field, and several new types of RNA molecules have been discovered, leading to the development of several studies designed to implement more effective treatments for diseases that still remain without any cure [40]. RNA-based therapeutics can be classified into five different categories: (1) mrna, which encodes for proteins; (2) siRNA, which are double-stranded and primarily cause translational repression of their target protein; (3) mRNAs, which are small RNAs that can either inhibit protein synthesis when they bind to an mRNA target (miRNA mimics) or free up mRNA by binding to the miRNA that represses the translation of that particular mRNA (miRNA inhibitors); (4) antisense oligonucleotides, which are small (~15–25 nucleotides) single-stranded RNAs that can either promote or repress target expression; and (5) aptamers, which are short single-stranded nucleic acids that form secondary and tertiary structures that inhibit several types of target molecules, including proteins [83,84,88].

**Figure 4:**
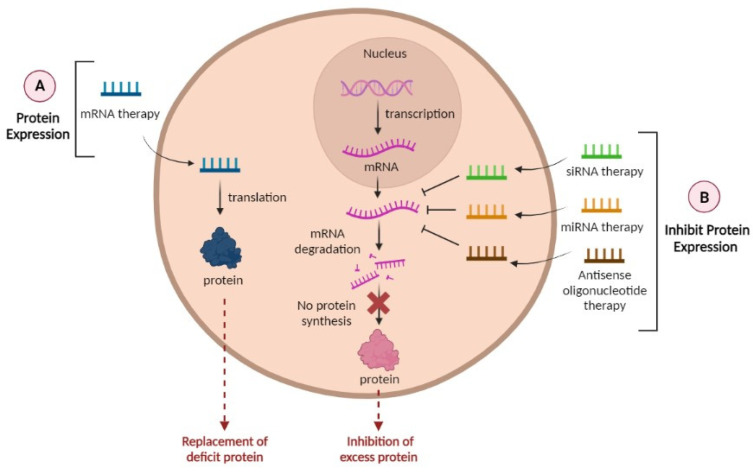
Possible RNA-based therapeutic strategies for AD. (**A**) mRNA leads to protein expression and (**B**) siRNA, miRNA, and antisense oligonucleotide inhibit protein expression. Created in BioRender.com (accessed on 20 April 2023).

The use of nucleic acid therapy has limitless potential to treat not only neurological diseases but also a considerable range of disorders since preclinical studies in cellular and animal models proved that mRNAs and short RNAs can be a new class of medicine [89]. Considering the unmet need for effective treatments for AD patients, it is crucial to evaluate diverse therapeutic targets and strategies to cure this disease [44]. In recent years, Aβ and Tau proteins had been the principal targets for researchers. Despite being well-documented hallmarks of AD, treatments involving the regulation of these proteins, unfortunately, are still an unsuccessful strategy [45,90]. In this review, we are going to focus on RNA therapies such as siRNA, miRNA, and mRNA for the treatment of AD (Figure 4) and describe some examples in Table 2.

- miRNA

miRNAs are small non-coding single-stranded RNAs approximately ~22 nucleotides in length [91]. Endogenous miRNAs are essential in cell development and play a key role in post-transcriptional gene regulation since they regulate the expression of multiple mRNAs both by promoting mRNA degradation and blocking the translation of multiple target mRNAs, inhibiting protein synthesis [84]. They can regulate mRNA translation by binding to the 3′untranslated region, allowing the reduction in the amount of target protein, instead of only inhibiting its activity [40,92]. In addition to gene expression regulation, miRNA can also act as signaling molecules for intercellular communication, revealing that it can be packaged into exosomes to exert this function [93]. The miRNA therapeutic strategy could be categorized into two types: miRNA mimics and miRNA inhibitors [5,82]. miRNA mimics are synthetic RNA molecules that are designed to act as endogenous miRNA to silence genes and can be applied when increased levels of mRNA are prevalent. In contrast, miRNA inhibitors are synthetic ssRNA molecules and can interrupt the miRNA function via sequence-specific binding to mature miRNA, without causing gene silencing. This option is interesting when protein synthesis restoration is needed [82]. These particular features allow miRNA to target multiple sites of various molecularly deregulated cascades in disease conditions, similar to what occurs in AD [40,91]. miRNAs molecules have crucial functions in the nervous system, such as neuronal differentiation, neurite outgrowth, and synaptic plasticity, and are responsive to neuropathological processes, including oxidative stress, neuroinflammation, and protein aggregation. This proves that miRNAs are key molecules in AD, and the dysfunction of miRNAs in this neurological disease is being recognized [45,91].

- siRNA

siRNAs are short, synthetic double-stranded RNA oligonucleotides (20–25 nt) that take advantage of the RNA interference pathway to silence gene expression by targeting their complementary mRNA [83,84]. siRNAs offer promising therapeutics for brain disease treatment by directly blocking causative gene expression with high targeting specificity, requiring low effective dosages, and benefiting from a relatively simple drug development process [94]. Several siRNA-based therapeutics were already approved by the FDA for other diseases, supporting their potential use for AD therapeutics [40].

- mRNA

mRNA is a type of single-stranded RNA involved in protein synthesis. The role of mRNA is to carry protein information from the DNA in a cell’s nucleus to the cell’s cytoplasm. Compared to other RNA therapies, mRNA can provide advantages such as (1) safety, (2) effectiveness, particularly in slowly dividing or non-dividing cells such as neural cells, and (3) better control of protein expression [40].

**Table 2:** miRNA, siRNA, and mRNA therapeutic applications in Alzheimer’s disease.

	Role in AD	References
**Types of miRNA**		
miR-101	Significantly reduced the expression of a reporter under control of APP 3′-UTR in HeLa cells.	[95]
miR-106b	Overexpression of miR-106b inhibited Aβ_1-42_-induced tau phosphorylation at Tyr18 in SH-SY5Y cells stably expressing Tau.	[96]
miR-137	miR-137 inhibited increased expression levels of p-tau induced by Aβ_1-42_ in SH-SY5Y and inhibited the hyperphosphorylation of Tau protein in a transgenic mouse model of AD.	[97]
miR-219	In a *Drosophila* model that produces human Tau, reduction of miR-219 exacerbated Tau toxicity, while overexpression of miR-219 partially annulled toxic effects.	[98]
miR-17	miR-17 inhibits elevated miR-17 in adult AD (5xFAD) mice microglia improves Aβ degradation.	[99]
miR-20b-5p	Treatment with miR-20b-5p reduced APP mRNA and protein levels in cultured human neuronal cells.	[100]
miR-29c	Over-expression of miR-29c in SH-SY5Y, HEK-293T cell lines and miR-29c in transgenic mice downregulated BACE1 protein levels.	[101]
miR-298	miR-298 is a repressor of APP, BACE1, and the two primary forms of Aβ (Aβ40 and Aβ42) in a primary human cell culture model. Thus, miR-298 significantly reduced levels of ~55 and 50 kDa forms of the Tau protein without significant alterations of total Tau or other forms.	[102]
miR-485-5p	miR-485-5p overexpression facilitated the learning and memory capabilities of APP/PS1 mice and promoted pericyte viability and prohibited pericyte apoptosis in this model.	[103]
miR-9-5p	miR-9-5p overexpression inhibited Aβ_25-35_-induced mitochondrial dysfunction, cell apoptosis, and oxidative stress by regulating GSK-3β expression in HT22 cells.	[104]
miR-132	miR-132 inhibited hippocampal iNOS expression and oxidative stress by inhibiting MAPK1 expression to improve the cognitive function of rats with AD.	[105]
miR-153	Using miR-153 transgenic mouse model, was verified that miR-153 downregulated the expression of APP and APLP2 protein in vivo.	[106]
**Targeted gene silencing by siRNA**		
Tau	siRNA against MAPT can effectively suppress tau expression in vitro and in vivo without a specific delivery agent.	[107]
BACE1	Polymeric siRNA nanomedicine targeting BACE1 in APP/PS1 transgenic AD mouse model can efficiently penetrate the BBB via glycemia-controlled glucose transporter-1–mediated transport, ensuring that siRNAs decrease BACE1 expression.	[94]
Presenilin1 (PS1)	Downregulation of PS1 and Aβ_42_ in IMR32 cells transfected with siRNA against PS1 was verified.	[108]
APP	Infusion of siRNAs that down-regulated mouse APP protein levels into the ventricular system for 2 weeks down-regulated APP mRNA in mouse brain.	[109]
**Proteins encoded by mRNA**		
mRNA encoding neprilysin	Neprilysin plays a major role in the clearance of Aβ in the brain. New mRNA therapeutic strategy utilizing mRNA encoding the mouse neprilysin protein has been shown to decrease Aβ deposition and prevent pathogenic changes in the brain.	[110]

### Overcoming Limitations of RNA Therapies with Delivery Systems

In spite of being a hot research topic in the present day, the development of novel RNA therapeutics has proven to be highly challenging in the past two decades. Some of the major disadvantages that stop RNA from being a clinical success are its instability for in vivo application since the human body has several intrinsic defense systems to protect the cells against exogenous molecules, such as ribonucleases (RNases). RNA’s low targeted tissue accumulation decreases its therapeutic efficacy, requiring high therapeutic RNA doses that can induce toxicity [111]. Early degradation of naked RNAs is another hurdle for their therapeutic efficiency since they tend to be promptly eliminated from the body via renal or hepatic clearance, shortly after the systemic administration [82,111]. For reference, the half-life of naked siRNA is approximately 15 min, and the half-life of naked mRNA can vary between 2 and 25 min, depending on the presence of 5′ capping, the length of the 3′ poly-A tail, and the RNA secondary structure [112]. Finally, being a large and negatively charged molecule, it is difficult to deliver into the cellular cytoplasm, where it exerts its action. Even if cellular internalization of RNA occurs, there is also the risk that RNA cannot escape the endosomal pathway, with only, for example, ~1–2% of siRNAs uptaken by the cells escaping the endosome [81,82].

**Figure 5:**
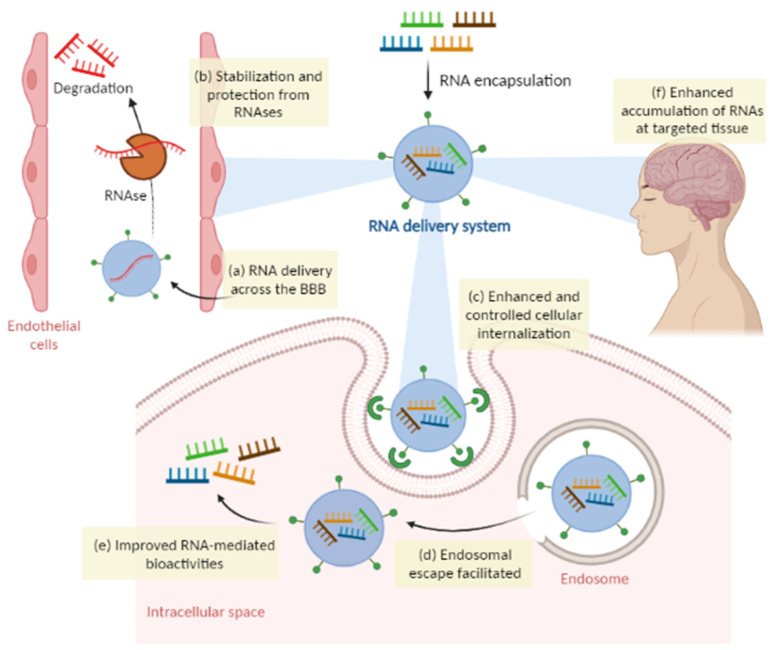
How RNA delivery systems can overcome the physiological obstacles faced by naked RNAs. (**a**) RNA can be delivered across the BBB; (**b**) protection of RNAs from degradation by ribonucleases (RNAses); (**c**) enhanced cellular internalization by controlled or specific pathways; (**d**) intracellular endosomal degradation can be avoided by endosomal escape; (**e**) improved RNA-mediated bioactivities; (**f**) enhanced accumulation of RNA in target tissue. Created in BioRender.com (accessed on 20 April 2023).

These obstacles have been considerably surpassed, thanks to the recent advancements in research areas such as RNA biology and nanotechnology that allowed the development of new materials and technologies for the delivery of RNA molecules [81,84]. These new advances transformed RNA technology into a novel therapeutic too since RNA can now be safely transported and delivered to the target thanks to delivery systems [82] (Figure 5).

RNA delivery is key for the treatment of diseases such as neurological disorders, as it gives RNA the ability to target diseases that cannot be treated with other conventional drug groups by encapsulating these molecules in delivery vectors. These RNA vectors have the capacity to effectively protect this molecule from biodegradation, increase bioavailability, solubility and permeation, surpass biological barriers, and promote targeted delivery and release. Hence, RNA nanoencapsulation potentiates the treatment of diseases by silencing genes or expressing therapeutic proteins [113,114].

## 5. Nanoparticles and the BBB

Delivery systems can overcome RNA’s limitations, becoming a successful therapy, particularly for facilitating the crossing of the BBB, which is the main culprit for the shortage of new and effective treatments for AD [115].

In this review, we will explain the BBB anatomic composition and characteristics, as well as the BBB pathways into the CNS. Subsequently, we will discuss exosomes, liposomes, and exosome-like liposomes as possible tools for RNA transport across the BBB.

**Figure 6:**
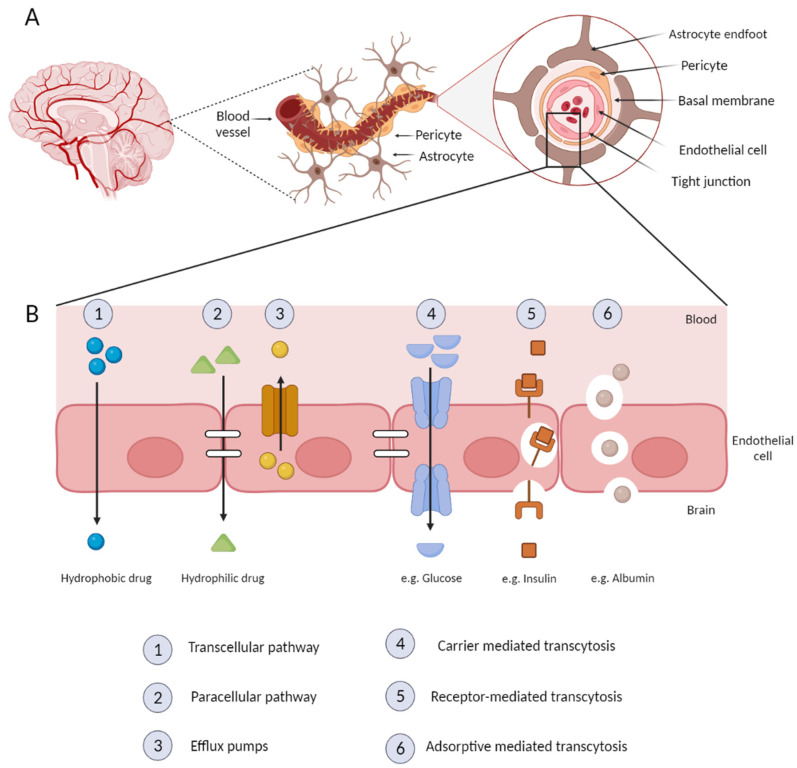
Structural representation of the BBB anatomy (**A**) and possible pathways of entrance into the central nervous system (**B**). Created in BioRender.com (accessed on 20 April 2023).

From an anatomical point of view, the BBB is composed of different cell types such as endothelial cells, pericytes, and astrocytes (Figure 6A) [116]. In between the endothelial cells, there are tight junctions, which are surrounded by a thin basal membrane and astrocytes vascular feet. These highly restrictive tight junctions are a key BBB feature since they are responsible for the barrier properties and limit the transfer of almost all drugs [116,117]. Pericytes cover 20% of the outer surface of endothelial cells and are responsible for the regulation of the blood flow in the brain capillary through contraction and relaxation. The astrocytes are glial cells that connect the brain capillary and neurons and also maintain BBB functions by providing nutrients to neurons and protecting the brain from oxidative stress and metal toxicity [117]. Additionally, the basal membrane provides structural support around the pericytes and endothelial cells [118].

This specialized barrier acts as an interface with the capacity to regulate the entry of plasma components, red blood cells, and leukocytes into the CNS and ensures the export of potentially neurotoxic molecules from the brain to the blood [119,120]. This barrier strictly controls the molecule movements between the blood and the brain, regulating the homeostasis of the nervous system [119]. Moreover, more than 98% of all small-molecule drugs and approximately 100% of biological drugs are incapable of crossing the BBB. Additionally, water-soluble molecules in the blood are prevented from entering the CNS, while lipid-soluble molecules are reduced by enzymes or efflux pumps [115,121].

The BBB possesses several permanently active transport mechanisms to ensure the transport of nutrients into the CNS while excluding blood-borne molecules that could be detrimental [122]. On one hand, these BBB properties are proof of its vital role in maintaining the specialized microenvironment of the brain tissue. On the other hand, these features make CNS access one of the most difficult of the body, limiting the development of novel effective drugs to treat AD [119]. Even though the BBB is a strict barrier for the circulation of molecules between the blood and the CNS, there are a few pathways that allow the delivery of essential molecules that maintain brain homeostasis (Figure 5B). These include the transcellular pathway, the paracellular pathway, efflux pumps, carrier-mediated transcytosis, receptor-mediated transcytosis, and adsorptive-mediated transcytosis [114].

In a healthy BBB, transcellular diffusion (Figure 6B1) consists of the diffusion of solute particles through the endothelial cells. Particles transported through this route are small lipophilic molecules that penetrate through the cells. On the other hand, paracellular transport (Figure 5B is restricted by the tight junctions between the endothelial cells, allowing only hydrophilic molecules to pass, with a molecular weight < 500 Da.

Efflux pumps (Figure 6B3) are a set of proteins responsible for limiting the accumulation of various potentially toxic molecules, and eventually, for expelling these molecules from the brain. These proteins are a limiting factor for the delivery of bioactive compounds to the brain.

Carrier-mediated transcytosis (Figure 6B4) consists of active transport with the support of carrier proteins such as the glucose transporter isoform (GLUT-1) and the large amino acid transporter (LAT), allowing entrance to glucose or amino acids. The transport of these molecules occurs when they bind to the protein on the blood side of the BBB, and a subsequent conformational change allows their transport into the brain [114,123].

Receptor-mediated transcytosis (Figure 6B5) is a specialized transport system by which endogenous molecules can cross the BBB through receptors present on the cell surface. This type of transport relies on the following mechanisms: Endocytosis, intracellular vesicular trafficking, and exocytosis. Active components bind to their specific receptors on the luminal side of the endothelial cells, and an intracellular vesicle is formed through membrane invagination. The formed vesicles cross the cell to release the ligand at the basolateral side via exocytosis. The most common receptors involved in this process are the transferrin receptor (TfR), the insulin and insulin-like growth factor receptors, the low-density lipoprotein receptor (LDLR), the low-density lipoprotein-receptor-related protein 1 and 2 (LRP1 and LRP2), the scavenger receptor class B type I (SR-B1), the leptin receptor, the albumin receptor, and the lactoferrin receptor. Receptor-mediated transcytosis is one of the most promising pathways for nanoparticle drug delivery through the BBB [114,122].

Adsorptive-mediated transcytosis (Figure 6B6) is another important BBB-crossing pathway, without the involvement of specific plasma-membrane receptors. The basic mechanism is responsible for the transport of charged particles by taking advantage of the electrostatic interactions between the positively charged drug carriers and the negatively charged luminal membrane of the brain endothelial cells. This transport has lower affinity but higher capacity compared to receptor-mediated transcytosis [114,122,123].

There is a crucial need for an ideal and safe approach to effectively carry pharmaceutical agents in a target-specific and sustained-release manner into the CNS, without disrupting the BBB [122,123]. A promising approach is taking advantage of receptor-mediated transcytosis for drug delivery to the brain with the help of ligand-functionalized nanoparticles. Nanoparticles are gaining popularity as drug carriers for the treatment of neurological disorders, due to their small size and unique physical properties [124]. By virtue of their biochemical composition, lipidic nanoparticles provide biomimicking and bio-degradable platforms. As a result of their lipophilicity and size, nanosized particles such as exosomes and liposomes are promising drug delivery carriers to increase the penetration of the BBB [113,125].

### 5.1. Exosomes

Exosomes are a subset of EVs with a diameter ranging from 30 to 100 nm, and their composition includes lipids, proteins, and nucleic acids [126,127]. Their lipid content includes sphingomyelin, phosphatidylserine, cholesterol, and ceramide or derivatives. Exosomes also carry non-specific proteins (e.g., cytoplasmic enzymes, cytosolic proteins, heat shock proteins, and transferring proteins) and specific proteins that differ from one exosome to another, depending on their origin. The genetic material of these vesicles includes microRNAs, mRNAs, long non-coding RNAs, and DNA fragments [126]. Exosomes are also enriched in late endosome components such as CD63, CD9, and CD81 since they originate from the endocytic compartment of the producer cell in a process that generates multi-vesicular endosomes (MVEs), which subsequently fuse with the plasma membrane to release exosomes into the extracellular space.

Exosomes yield information that can reflect the phenotype of the parental cell since these vesicles carry distinct RNA and protein cargoes that allow the identification of their parental cells, as well as cell-specific or tissue-specific factors that can be used to determine their site of origin [128,129]. Moreover, when exosomes are secreted, undesirable proteins and other molecules are discarded, making these vesicles a compartment of cellular debris for subsequent disposal [130].

Originally, exosomes were primarily described as being for the elimination of excessive and unnecessary molecules from the cells. However, in the last decade, it has been shown that they have other key functions in both physiological and pathological processes. Regarding the physiological roles, exosomes play an important role in intercellular communication since they are able to deliver a number of bioactive cargos to near or distant target cells [126]. Moreover, exosomes display a role in tissue homeostasis and have anti-inflammatory functions. An example of their function is neuronal communication via the secretion of exosomes, which can contribute to a range of neurobiological functions, including synaptic plasticity. In relation to pathological functions, they control the expansion and progression of diseases, such as cancer and neurodegenerative diseases [130]. Exosomes are involved in the complex mechanisms of secretion, spread, and degradation of the Aβ and Tau proteins and are, especially, involved in Tau propagation between neuronal cells [131].

The unique properties of exosomes include their small size, durability, stability, potential cell selectivity, low immunogenicity, and ideal biocompatibility. These properties make them good candidates to be a therapeutic delivery system. Compared to traditional therapeutic drugs, exosomes have a higher potential to pass through the BBB, which helps the drugs they might carry to reach the CNS. Moreover, since exosomes can be isolated from all body fluids, they can be candidates for analysis as part of a non-invasive liquid biopsy [129,130,131].

### 5.2. Liposomes

In the last two decades, lipid-based nanoparticles (LNPs), especially liposomes, have been attractive nanometric delivery systems for being the most well-studied nonviral platforms for the delivery of RNA molecules and achieving significant clinical success [113,132] highlighted by the highly effective mRNA COVID-19 vaccines [132,133].

There are several types of LNPs such as liposomes, niosomes, transfersomes, nanoemulsions, solid lipid nanoparticles (SLNs), lipid nanocapsules (LNCs), nanostructured lipid carriers (NLCs), lipid-based micelles, core–shell lipid nanoparticles (CLNs), and hybrid lipid-polymeric nanoparticles [132,134]. LNPs range in size from 40 nm to 1000 nm and are colloidal lipophilic systems constituted by four main components: A pH-sensitive cationic lipid, a helper lipid, cholesterol, and a PEG-lipid. The cationic lipid is a synthetic lipid, constituted by a hydrophilic head with a protonable tertiary amino group (pKa 6–6.7) and a long hydrophobic tail. Cholesterol is incorporated into the LNPs formulation with the goal of increasing their flexibility, whilst the helper phospholipid assists in the process of endosomal escape and contributes to the stability of LNPs [135,136]. Finally, the insertion of a short-chain PEG-lipid derivative (normally of 14 carbon atoms) is essential to maximize the ex vivo stability and control the particle size before administration [137].

Due to their resemblance to biological and natural components, these nano systems show tremendous promise as carriers for therapeutic applications. The main advantages of LNPs over other nanoparticles are their low toxicity and biocompatibility, biodegradability, safety, high mechanical and chemical versatility, and the capacity to protect the active ingredient from degradation processes induced by external factors. Along with these features, these lipid-based nanoparticles can incorporate the delivery of both hydrophobic and hydrophilic molecules and most of their preparation methods can be easily scaled up. Additionally, because of their lipophilicity, LNPs possess the ability to overcome difficult physiological barriers, such as the BBB, even without surface modification [124,134].

Among all the nano-based drug delivery systems, liposomes are the most biocompatible and least toxic since they are composed of phospholipids and cholesterol, the main components of cell membranes. Liposomes are an extremely versatile nanocarrier platform that has the capacity to load multiple drugs, provide protection from degradation, have controlled and targeted drug release, and enhance drug endocytosis into cells [11]. Additionally, these nanoparticles are able to incorporate both hydrophilic and hydrophobic therapeutic agents. The hydrophilic compounds may either be entrapped into the aqueous core of the liposomes or be located at the interface between the lipid bilayer and the external water phase, while the hydrophobic compounds are generally entrapped in the hydrophobic core of their lipid bilayers. The positively charged lipids in the liposome’s constitution allow the electrostatic interaction with negatively charged nucleic acids, such as DNA and RNA, in gene delivery applications [138].

There is evidence that lipid-based nanoplatforms will play a key role in the development of RNA neuro-therapies, with liposomes being one of the main lipidic platforms for RNA delivery to the CNS [113]. In order to enhance drug delivery into the CNS, the liposome surface can be modified by the inclusion of biologically active ligands, such as peptides, polysaccharides, antibodies, or aptamers, which specifically bind to receptors expressed on the surface of the brain endothelial cells, facilitating their binding and transport across the BBB. The addition of polyethylene glycol (PEG) offers superficial protection for the liposomes by avoiding binding with plasma proteins, and hence, preventing their opsonization and subsequent clearance. PEGylation of liposomes prolongs their circulation time in the body and also plays a crucial role in brain drug delivery, allowing liposomes to cross the BBB [138].

### 5.3. Exosome-like Liposomes as a Novel Strategy

Scientists have explored various nanomaterials for targeted delivery through the BBB with significant efficacy, such as dendrimers, polymeric nanoparticles, gold nanoparticles, carbon quantum dots, and exosome-like liposomes [8,139]. First, in this review, we will summarize the exosome-like liposomes’ competition and then focus only on this delivery system.

- Dendrimers: Dendrimers are highly branched, characterized by defined molecular weights and specific encapsulation properties. This type of delivery system is composed of symmetrical polymeric macromolecules with a large number of reactive surface groups, with three distinctive architectural components: An interior core, an interior layer consisting of repeating units radially attached to the inner core, and functional end groups on the outside layer. Because of these unique features, dendrimers can cross-impair the BBB and target astrocytes and microglia after systemic administration in animal models [139].
- Polymeric nanoparticles: Polymeric nanoparticles can be produced from synthetic or natural polymers. However, to be applied in brain drug delivery, these nanoparticles need to be biodegradable and biocompatible. PBCA, PLA, and PLGA nanoparticles are nanoparticles able to cross the BBB. These nanocarriers possess controlled drug release, targeting efficiency, and can avoid phagocytosis by the reticuloendothelial system, thus improving the concentration of drugs in the brain [140].
- Gold nanoparticles: Nanoparticles (mostly < 10 nm in size) composed of a gold core and with covalently or non-covalently attached surface ligands. Multiple in vivo studies on rodents have shown that low amounts of this delivery system were able to cross the BBB. However, greater amounts of the administered dose were found in the liver and in the blood [8]. Additionally, Sela et al. proved that gold nanoparticles could penetrate the BBB of rats without the use of an external field or surface modification and were found to be distributed uniformly in both the hypothalamus and hippocampus indicating there is no selective binding in these regions of the brain [141].
- Carbon quantum dots: This delivery system retains a polymeric core structure and various functional groups on the surface, facilitating their conjugation with drug molecules for specific delivery. This is a carrier with several efficient features for BBB crossing such as excellent biocompatibility and low toxicity due to the lack of metal elements, small size, and photoluminescence, which can be utilized to track the penetration of CDs through the BBB [117].

**Figure 7:**
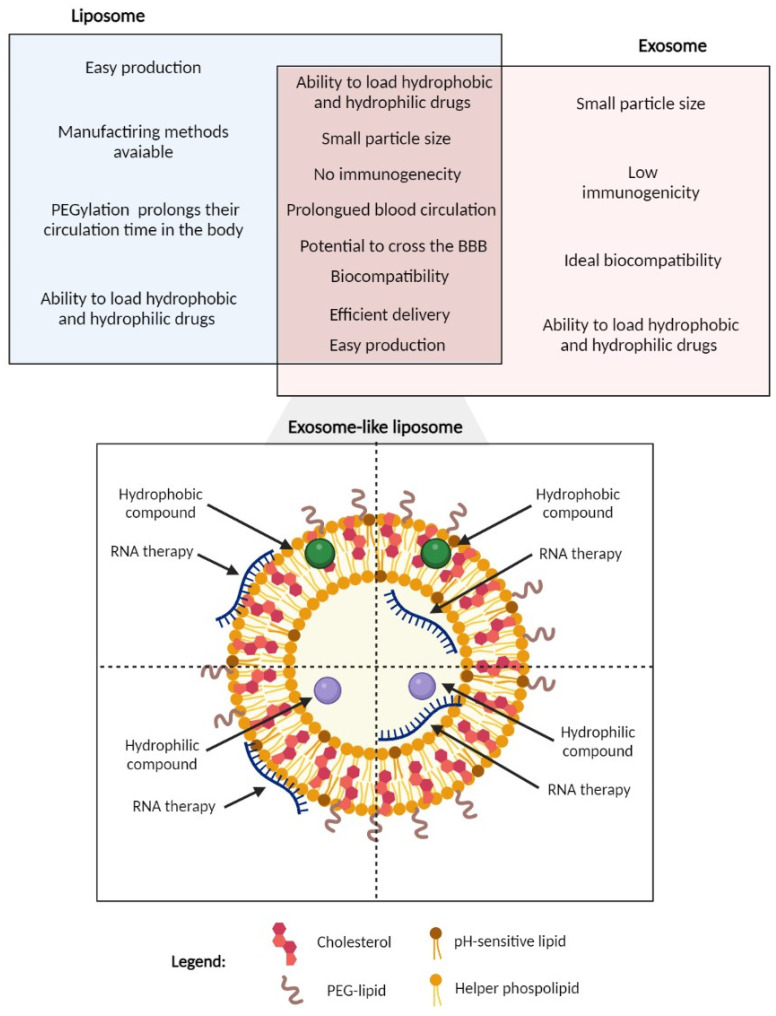
Exosome-like liposomes combine advantages of liposomes and exosomes, with the capacity to be loaded with different types of molecules due to their constitution. Created in BioRender.com (accessed on 20 April 2023).

In addition to the fact that these nanoparticles are promising in overcoming the BBB, exosome-like liposomes are also delivery systems with unique properties to achieve this goal. Exosomes and liposomes are promising nanocarriers with unique properties. However, these delivery systems have some limitations. For instance, liposomal targeting efficiency is limited, and they can induce immunogenicity [11], whilst efficient and reliable isolation and purification are needed for the clinical application of exosomes. It is also crucial to identify appropriate strategies to increase loading capacity and specificity to use exosomes as a nanocarrier [10,142]. When comparing the advantages and disadvantages of exosomes and liposomes, it becomes evident that the two systems are complementary, since the advantages of the one system mitigate the disadvantages of the other, and vice-versa (Figure 7). Therefore, the development of a system that incorporates the desirable features of the two carriers into one hybrid delivery system, led to an innovative carrier for drug delivery applications, the exosome-like liposome (Figure 7) [11,143]. These novel exosome-like liposomes are formulated with a lipid composition that mimics that of exosomes, which impart them with some of the desirable characteristics of exosomes, such as enhanced passive targeting, biocompatibility and RES evasion, and the ability to cross biological barriers, whilst allowing much higher encapsulation efficiencies and larger-scale production in good manufacturing practice that the use of exosomes themselves does not allow.

Thus, particle size plays a major role in drug delivery efficacy, including in the therapeutic effect achieved. The endothelial cells’ slit width is 200 to 500 nm. Consequently, for the long-term circulation of these nanoparticles, they need to have a size that does not exceed 200 nm, leading to better brain drug delivery across the BBB. Moreover, the fact that exosome-like liposomes are coated with this suitable hydrophilic polymer leads to advantages such as escaping phagocytosis, avoiding opsonization, and further increasing the blood circulation time, since the hydrophobic nature of the nanoparticle containing the drug is shielded. These unique characteristics drive researchers to believe that this type of carrier can have a key impact in crossing the BBB, helping in the therapy of neurodegenerative diseases such as AD [123].

Additionally, exosome-like liposomes were highly efficient at encapsulating curcumin, with encapsulation efficiencies ranging between 85 and 94%, and were effective at delivering curcumin into neuronal cells to promote a superior neuroprotective effect after oxidative insult compared to free curcumin at the same concentrations. Thus, these delivery systems have shown to be highly biocompatible, without significantly affecting cell viability or causing hemolysis. Finally, the exosome-like liposomes have been shown to be internalized by zebrafish embryos and accumulate in lipid-rich zones, such as the brain and yolk sac, also in stages where the BBB is already formed [144]. In Table 3, we demonstrate other crucial examples of applications of exosome-like liposomes in vivo and in vitro.

## 6. Conclusions

AD is a neurodegenerative disease that has a tremendous impact on people’s quality of life, all around an aging world. This burden is further impacted by the lack of therapies, due to the complex mechanisms that lead to the disease onset and also due to limitations that the BBB imposes on the entrance and maintenance of therapeutic molecules in the CNS.

However, the emergence of RNA as an especially versatile tool for the treatment of several diseases has opened multiple new possibilities for the development of effective treatments for AD.

The accelerated growth of RNA therapies requires the development of efficient delivery systems to transport a selectively deliver the RNA molecules into their target cells or tissues, due to the low bioavailability of these molecules.

Nanoparticles have provided new and safer avenues for the delivery of natural compounds and RNA. In this field, novel exosome-like liposomes are positioning themselves as a prime solution for the vehiculation of therapeutical compounds to the CNS, due to the combination of the favorable characteristics of exosomes with those of liposomes, which entitle scalable production in good manufacturing practices, which is currently unfeasible for the exosomes themselves.

## 7. Patents

The authors are co-inventors in a patent application (EXOSOME-MIMETIC LIPOSOME COMPOSITION AND USE, Instituto Nacional da Propriedade Industrial PPP No. 116560 P, 3 July 2020).

## Figures and Tables

**Table 1:** Natural compounds or extracts that can be used for AD therapy.

Natural Compound	Role in AD	References
Eugenol	Rats were fed aluminum, a neurotoxic metal that leads to oxidative brain injury and enhanced lipid peroxidation, disruption of neurotrophic, cholinergic, and serotonergic functions, and induce apoptosis with ultimate neuronal and astrocyte damages. A neuroprotective role of eugenol against the aluminum effects was verified through its antioxidant, antiapoptotic potential and its neurotrophic properties.	[51]
Menthol	Menthol inhalation by mice (1 week per month, for 6 months) prevented cognitive impairment in the APP/PS1 mouse model of Alzheimer’s.	[52]
Chrysin	Chrysin showed the ability to act as a membrane shield against early oxidative events mediated by O2˙- and other ROS that contribute to neuronal death triggered by AlCl_3_ exposure, showing chrysin’s neuroprotective action.	[53]
Rosmarinic acid	Suppresses Aβ accumulation in mice.	[54]
*Ginkgo biloba*	Ginkgo biloba improves microcirculation, inhibits the expression of inflammatory factors, and reduces inflammatory damage to neurons, thereby improving the spatial exploration memory of dementia model rats.	[55]
Resveratrol	Multiple studies demonstrated that resveratrol has neuroprotective, anti-inflammatory, and antioxidant characteristics and the ability to minimize Aβ peptide aggregation and toxicity in the hippocampus of Alzheimer’s patients, stimulating neurogenesis and inhibiting hippocampal degeneration. Furthermore, resveratrol’s antioxidant effect promotes neuronal development by activating the silent information regulator-1, which can protect against the detrimental effects of oxidative stress.	[56]
Huperzine A	Huperzine A is natural, potent, highly specific reversible inhibitor of acetylcholinesterase, with the ability to cross the BBB.	[57]
Brahmi	The neuroprotective properties of Brahmi include the reduction of ROS and neuroinflammation, the inhibition of the aggregation of Aβ and the improvement of cognitive and learning behavior.	[58]
*Uncaria tomentosa*	Inhibits plaques and tangles formation.	[59]
Berberine	Berberine has antioxidant activity and promotes AChE and monoamine oxidase inhibition. Berberine has been shown to improve memory, lower Aβ and APP concentration, and diminish Aβ plaque accumulation.	[60]
Quercetin	Behavioral and biochemical tests confirm that quercetin promotes the reduction in oxidative stress and increased cognition in zebrafish AD models induced with aluminum chloride.	[61]
Betaine	Betaine has been shown to decrease homocysteine levels and Aβ toxicity in *Caenorhabditis elegans* AD model.	[62]
Curcumin	Curcumin is known to be a potent antioxidant, anti-inflammatory and anti-amyloidogenic compound, that plays a beneficial role in treating AD through several mechanisms. Curcumin can promote a significant reduction of Aβ oligomers and fibril formation.	[46]
Crocin	Crocin, the main constituent of *Crocus sativus* L., has a multifunctional role in protecting brain cells, modulating aggregation of Aβ and Tau proteins, attenuating cognitive and memory impairments, and improving oxidative stress.	[63]
*Withania somnifera*	Withania somnifera extract can protect against Aβ peptide- and acrolein-induced toxicity. Treatment with this extract significantly protected against Aβ and acrolein, in various cell survival assays with the human neuroblastoma cell line SK-N-SH, significantly reduced the generation of ROS and was demonstrated to be a potent inhibitor of AChE activity.	[64]
*Poncirus trifoliate*	The extract of *Poncirus trifoliate* is a naturally occurring AChE inhibitor. It showed a 47.31% inhibitory effect on the activity of acetylcholine.	[65]
*Convolvulus pluricaulis*	*Convolvulus pluricaulis* prevented aluminum-induced neurotoxicity in rat cerebral cortex.	[66]
α-Cyperone	α-Cyperone binds and interacts with tubulin, being capable of destabilizing microtubule polymerization. The effect of this interaction could result in reduction of inflammation.	[67]
Andrographolide	Andrographolide has beneficial effects in the recovery of spatial memory and learning performance, recovery of synaptic basal transmission, partial or complete protection of certain synaptic proteins and shows a specific neuroprotective effect, that includes the reduction of phosphorylated Tau and Aβ aggregate maturation, in aged degus.	[68]
Apigenin	Apigenin has been shown to have anti-inflammatory and neuroprotective properties in a number of cell and animal models. This compound is also able to protect human induced pluripotent stem cell-derived AD neurons via multiple pathways, by reducing the frequency of spontaneous Ca^2+^ signals and significantly reducing caspase-3/7 mediated apoptosis.	[69]
Baicalein	Baicalein has antioxidant and anti-inflammatory effects.	[70]
Carvacrol	Carvacrol possesses anti-AChE, antioxidant, and neuroprotective properties. This compound alleviated Aβ-induced deficits by reducing cellular neurotoxicity and oxidative stress in the SH-SY5Y cell line, and by reducing oxidative stress and memory impairment in a rat model of AD.	[71]
Decursin/Decursinol angelate	Decursin and decursinol angelate increase cellular resistance to Aβ-induced oxidative injury in PC12 cells.	[72]
Genistein	In vivo studies have shown that genistein improves brain function, antagonizes the toxicity of Aβ and has neuroprotective effects.	[73]
Wogonin	Wogonin has various neuroprotective and neurotrophic activities, such as inducing neurite outgrowth.	[74]
Rutin	Rutin is antioxidant, anti-inflammatory, and has the capacity of reducing Aβ oligomer activities.	[75]
Luteolin	Luteolin has the capacity to cross the BBB and can inhibit β- and γ-secretase to decrease Aβ. It can also reduce neuroinflammation and attenuate the phosphorylation of Tau.	[76]
Linalool	A linalool-treated mice model of AD showed improved learning and spatial memory. This compound reverses the histopathological hallmarks of AD and restores cognitive and emotional functions via an anti-inflammatory effect.	[77]
Asiatic acid	Pre-treatment with Asiatic Acid enhanced cell viability, attenuated rotenone-induced ROS, mitochondrial membrane dysfunction and apoptosis regulating AKT/GSK-3β signaling pathway, after aluminum maltolate neurotoxicity induction in SH-SY5Y neuroblastoma cells.	[78]

**Table 3:** Examples of applications of exosome-like liposomes.

Exosome-like Liposomes Applications	References
Exosome mimetics-mediated gene-activated matrix encapsulating the plasmid of vascular endothelial growth factor (VEGF) was able to sustainably deliver *VEGF* gene and significantly enhance the vascularized osteogenesis in vivo.	[145]
PSMA-exosome mimetics showed increased cellular internalization in PSMA-positive PC cell lines (LNCaP and C4-2B) and higher tumor targeting was observed in solid C4-2B tumors, following intravenous administration, confirming their targeting ability in vivo.	[146]
Exosome mimetics are reported for bone targeting involving the introduction of hydroxyapatite-binding moieties through bioorthogonal functionalization. Bone-binding ability of the engineered exosome mimetics is verified with hydroxyapatite-coated scaffolds and an ex vivo bone-binding assay.	[147]
Administration of mesenchymal stem cells-exosome mimetics in conjunction with an injectable chitosan hydrogel into mouse nonhealing calvarial defects demonstrated robust bone regeneration.	[148]
Bone marrow mesenchymal stem cells were sequentially extruded to generate exosome-mimetic to encapsulate doxorubicin to treat osteosarcoma. The results showed that demonstrated significantly more potent tumor inhibition activity and fewer side effects than free doxorubicin.	[149]
In vitro, chemotherapeutic drug-loaded exosome-mimetics induced TNF-R-stimulated endothelial cell death in a dose-dependent manner. In vivo, experiments in mice showed that the chemotherapeutic drug-loaded exosome-mimetics traffic to tumor tissue and reduce tumor growth without the adverse effects observed with equipotent free drug.	[150]
Multifunctional exosomes-mimetics decorated with angiopep-2 (Ang-EM) incorporating Docetaxel, for enhancing glioblastoma drug delivery by manipulating protein corona, Ang-EM showed enhanced BBB penetration ability and targeting ability to the gioblastoma. Ang-EM-mediated delivery increased the concentration of docetaxel in the tumor area.	[151]
A designed lung-targeting liposomal nanovesicle carrying miR-29a-3p that mimics the exosomes, significantly down-regulated collagen I secretion by lung fibroblasts in vivo, thus alleviating the establishment of a pro-metastatic environment for circulating lung tumor cells.	[152]

## Data Availability

Data will be made available upon request.
